# Current Trends in Clinical Characteristics, Diagnosis, and Treatment of Polypoidal Choroidal Vasculopathy: A Perspective from Vietnam

**DOI:** 10.3390/jcm11164678

**Published:** 2022-08-10

**Authors:** Dang Tran Dat, Nguyen Do Thi Ngoc Hien, Nguyen Nhu Quan, Mai Quoc Tung, Hoang Chi Tam, Bui Viet Hung

**Affiliations:** 1 Outpatient Department, Vietnam National Eye Hospital, 85 Ba Trieu, Nguyen Du Ward, Hai Ba Trung District, Hanoi 100000, Vietnam; 2 Department of Ophthalmology, Hanoi Medical University, 1 Ton That Tung Street, Hanoi 100000, Vietnam; 3 Phuong Dong International Eye Center, 71 Ngo Thoi Nhiem Street, District 3, Ho Chi Minh City 700000, Vietnam; 4 Ophthalmology and Refractive Surgery Department, FV Hospital, 6 Nguyen Luong Bang Street, Phu My Hung, District 7, Ho Chi Minh City 700000, Vietnam; 5 Vitreoretial Department, Vietnam National Eye Hospital, 85 Ba Trieu, Nguyen Du Ward, Hai Ba Trung District, Hanoi 100000, Vietnam

**Keywords:** polypoidal choroidal vasculopathy, Vietnam, anti-VEGF, neovascular age-related macular degeneration, laser therapy

## Abstract

Polypoidal choroidal vasculopathy (PCV) is a common choroidal disease in the Asian population including Vietnam and is characterized by subretinal red-orange nodules, pigmented epithelium detachment, and massive subretinal hemorrhage. The recent focus on PCV in Vietnam can be attributed to advancements in PCV diagnosis and treatment. However, there is a scarcity of published literature and clinical data on PCV in the Vietnamese population, highlighting a key knowledge gap in this region. In order to address this gap, we gathered the opinions of experienced clinicians and retinal experts in Vietnam and reviewed available medical literature with the aim of: (i) providing an overview of PCV in the Vietnamese population—in terms of epidemiology, clinical characteristics, and management; (ii) tailoring international/national guidelines for the diagnosis and management of PCV, in line with available resources and medical equipment in Vietnam; and (iii) identifying gaps in clinical data in order to guide future PCV research in Vietnam and other countries with similar clinical conditions. The present review will enable healthcare providers and researchers to gain insight into current clinical practices and the limitations of PCV management in Vietnam and provide optimal and effective solutions.

## 1. Introduction

Polypoidal choroidal vasculopathy (PCV), a subtype of neovascular age-related macular degeneration (nAMD), is the leading cause of irreversible blindness globally. Overall, age-related macular degeneration (AMD) accounts for 8.7% of blindness globally, primarily affecting developed nations [1,2]. The prevalence of PCV among patients with nAMD is high in Asian countries, ranging from 24.5% to 54.7% [3]. PCV manifests as type 1 neovascularization associated with an abnormal branching vascular network or pachychoroid [4,5]. The nomenclature of aneurysmal type 1 neovascularization has recently been suggested as an alternative to PCV [5]. However, the group of Asian experts then issued a consensus-based recommendation not to use the term [6]. Yannuzzi et al. were the first to describe PCV as polypoidal, subretinal, vascular lesions with serious and hemorrhagic detachments, and to call it idiopathic PCV [7]. The presence of orange nodules and recurrent serosanguineous maculopathy are the primary clinical characteristics of PCV [4]. Apart from blindness, PCV is also associated with multiple recurrent hemorrhages [8], pachyvessels [9], retinal pigment epithelium detachment (PED) [10], and choroidal excavation [11].

While data on the prevalence and burden of PCV and retinal disease on patients in other parts of Asia are available in the medical literature, such information is lacking in Vietnam. Nonetheless, a few studies conducted in Vietnam have examined the clinical characteristics and risk factors associated with PCV. The Vietnamese Ministry of Health (MoH) has laid down guidelines for the diagnosis and management of choroidal retinopathies [12], but the current PCV management scenario in Vietnam has diverse limitations, such as the nonavailability of adequate diagnostic tools, applicability issues of anti-vascular endothelial growth factor (anti-VEGF) and photodynamic therapy (PDT) for treatment, and a lack of education and awareness among patients and healthcare providers. Therefore, the current review aims to highlight key knowledge gaps pertaining to the PCV landscape in Vietnam.

The objectives of this narrative review include: (i) providing an overview of PCV in the Vietnamese population—in terms of epidemiology, clinical characteristics, and management; (ii) tailoring international/national guidelines for the diagnosis and management of PCV, in line with available resources and medical equipment in Vietnam; and (iii) identifying gaps in clinical data to guide future PCV research in Vietnam and other countries with similar clinical conditions.

## 2. Methodology

The PubMed database was searched using different keywords, including “Polypoidal choroidal vasculopathy”, “Polypoidal choroidal vasculopathy” AND “diagnosis”, “Polypoidal choroidal vasculopathy” AND “treatment” OR “management” for relevant literature. The search was primarily focused on available health literature relevant to PCV in Asian countries, including Vietnam. The search was restricted to Vietnamese and English articles. Data sources were also searched to extract relevant literature pertaining to Vietnam, including data from the Data Integration Center of the Vietnamese MoH, the Vietnamese Inter-Library System, the libraries of medical universities in Vietnam, and the gray literature (government reports, conference proceedings, graduate dissertations, unpublished clinical trials) relevant to PCV. Published research and review articles obtained from the literature search were examined by a panel of practicing ophthalmologists from Vietnam to elucidate the PCV landscape in terms of epidemiology and clinical characteristics, and to highlight the key gaps in the diagnosis and clinical management of PCV in Vietnam.

## 3. Epidemiology and Clinical Characteristics

### 3.1. Prevalence

The clinical management of PCV has witnessed several advancements recently in diagnosis and therapy owing to the increasing global prevalence and disease burden of PCV. A meta-analysis conducted by Wong et al. revealed the overall global prevalence of AMD to be 8.69%, while the pooled global prevalence of early and late AMD was 8.01% and 0.37%, respectively [1]. In general, AMD was more prevalent in the Caucasian than the Asian population [1]; however, the Asian population is more prone to developing PCV than the Caucasian population [13,14,15]. The prevalence pattern of nAMD and PCV varied across Asia. In patients with nAMD, the prevalence of PCV ranged from 23 to 54.7% in Japan [16,17,18], to 49% in Taiwan [19] and 9.3–22.3% in China [20,21]. Additionally, variations in the clinical characteristics of PCV, based on ethnicity and racial differences, have been reported [15]. Currently, there are no population-based epidemiological studies on PCV in Vietnam. According to a national survey in Vietnam, the prevalence of PCV in patients with nAMD was 43.2% [22].

### 3.2. Demographics

The average age group of patients with PCV ranges from 50 to 65 years globally, with the mean age being 60.1 years, although the average age varied across Asia (Korea: 64.6 years, Japan: 72.8 years, China: 65.4 years, India: 60.06 years) and among Caucasians (75.4 years) [23]. In Vietnam, the mean age of patients with nAMD was found to be 67.6 years; however, the mean age of patients with PCV was lower, at 60.5 years [24,25]. In a nationwide survey conducted among Vietnamese ophthalmologists, PCV was found to be the most prevalent in the age groups of 51 to 60 and 61 to 70 years [22]. Previously, it was thought that the occurrence of PCV in female patients was more frequent [13]; however, more recently, it has been established that the proportion of male patients being affected by PCV within the Asian population is accelerating, with a higher prevalence noted in male patients compared to female patients [26]. However, the sex distribution within Vietnam for patients with PCV is quite balanced between male and female patients (1:1) [24].

### 3.3. Risk Factors

Certain studies, primarily for the Asian population, have demonstrated smoking as a critical risk factor in aggravating PCV. Studies show that smokers have a higher risk of PCV than those who do not smoke [27,28]. Smoking affects the optical density of the macular pigment and causes oxidative stress, leading to deterioration of the retinal pigment epithelium [29,30]. Alcohol consumption could also be regarded as a risk factor for PCV [31]. Cardiovascular and metabolic disorders, including hypertension (41–45% of patients with PCV), diabetes, and hyperlipidemia, have been shown to be associated with the incidence of PCV [23,27,28]. In patients with PCV, hypertension was associated with an almost 4-fold greater risk of developing recurrent subretinal hemorrhage [32]. Elevated levels of C-reactive protein are also a risk factor for PCV, suggesting the involvement of inflammatory processes in the pathomechanism of PCV [31]. Central serous chorioretinopathy and choroidal thickening are the other ocular risk factors commonly seen in patients with PCV [27,33].

Currently, in the Vietnamese context, there is a paucity of controlled studies evaluating the risk factors of PCV. In a meta-analysis, hypertension was identified as an important risk factor, where the prevalence of hypertension in the Vietnamese population ranged from 18.4 to 21.1% [34]. A study by Hien et al. involving Vietnamese patients with PCV observed that 36.6% of patients were smokers, 65.9% had hypertension, 22% had dyslipidemia, and 9% had diabetes [24]. Vietnamese patients with nAMD showed prevalence rates for smoking, hypertension, dyslipidemia, and diabetes in patients with PCV of 35%, 32%, 11% and 9%, respectively [25]. Vietnam has the third highest smoking rate in Southeast Asia, with smoking being higher in males (30.7%), which indicates that the Vietnamese population is at a higher risk of PCV [35]. According to a national survey of patients with PCV conducted in Vietnam, 48.5% and 56.7% of survey respondents perceived smoking and hypertension to be common risk factors for PCV, respectively. The proportion of ophthalmologists choosing diabetes mellitus and overweight as common risk factors for PCV was 35.1% and 10.3%, respectively [22]. 

### 3.4. Clinical Manifestations

**Figure 1:**
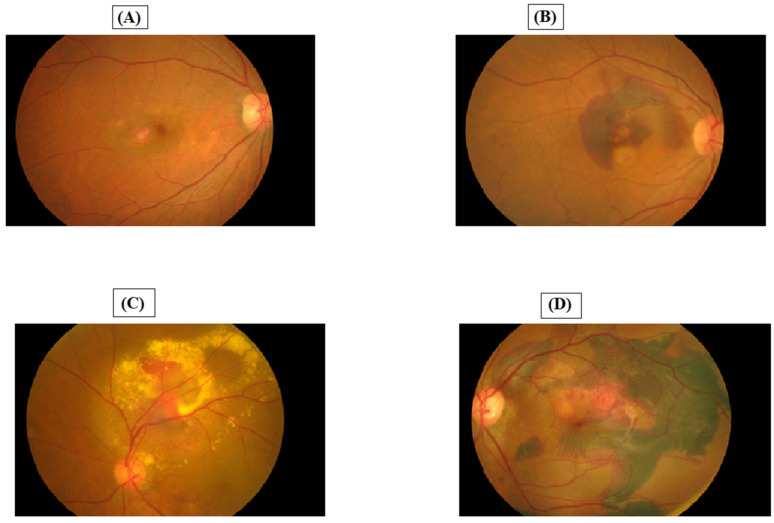
Various presentations of PCV on fundus photographs of Vietnamese patients (cases confirmed by ICGA). ((**A**) Two orange-red nodules, one at fovea and one in perifoveal region; (**B**) Multiples orange-red nodules at the macula with subfoveal hemorrhage; (**C**) Orange-red nodules at the upper vessel arcade, hard exudates and subretinal fluid; (**D**) Subfoveal orange-red nodules with extensive subretinal hemorrhage).

Significantly reduced visual acuity (20/200 [46.5% of patients]; >20/60 [14% of patients]), blurred vision (95.4%), dark spots (93%), and distorted vision (74.4%) are common clinical features in the Vietnamese population. However, the presence of yellow-orange nodules was found in only 53.5% of Vietnamese patients [24]. From the perspective of clinical ophthalmologists in Vietnam, the most common clinical signs indicative of PCV included orange nodules, subretinal hemorrhage, and pigment epithelial detachment [22]. Figure 1 shows the presentations of PCV on fundus photographs of Vietnamese patients (cases confirmed by ICGA). 

The results obtained from optical coherence tomography (OCT) showed that PED appeared in most patients with PCV (97.7%). Sharp-peaked PED is a common characteristic observed in Vietnamese patients (62.8%) [22]. In Vietnam, polypoidal lesions were found in the macula in 70% of patients with PCV [24], whereas in the Caucasian population, polypoidal lesions were found unilaterally (78.9%), bilaterally (25%), or extramacularly (47.3%) [36,37]. In the national survey of Vietnam, 88.7% and 69.1% of Vietnamese ophthalmologists confirmed the presence of polyps at the juxtafoveal and subfoveal sites, respectively [22].

### 3.5. Discussion and Expert Opinion

There is a big gap in epidemiological data for PCV in Vietnam, and hence, further research is warranted in this area. It can be assumed that the prevalence of PCV in Vietnam would not differ significantly from the reported prevalence in other Asian countries. Data pertaining to the Vietnamese population will enable healthcare authorities to plan public resources efficiently. With adequate infrastructure, human resources, equipment, and training, PCV diagnosis and treatment in Vietnam can be improved toward better overall clinical outcomes.

## 4. Diagnosis

The Vietnam MoH issued guidelines and recommendations in 2019 for the diagnosis and treatment of common vitreoretinal diseases, including PCV. Indocyanine green angiography (ICGA) is considered the gold standard for the diagnosis of PCV. However, in the absence of ICGA, PCV can still be diagnosed with 95% sensitivity and specificity when ≥2 of the following four criteria are present: V-shaped or hemorrhagic PED; sharp-peaked PED; V-shaped or multi-lobed PED; and hyper-reflective ring underneath PED detected using OCT [12]. An international expert group from the Asia-Pacific Ocular Imaging Society PCV Workgroup (APOIS) has released a consensus that outlines nine non-indocyanine green angiography (IGCA) criteria to diagnose PCV, wherein seven are based on OCT and two use colored fundus photography (FP) [6].

The PCV diagnostic criteria in Vietnam are based on criteria from the Japanese PCV research group and the criteria followed in the EVEREST study [24]. In routine practice, the combination of clinical assessment with imaging results is used for diagnosis in Vietnam. The clinical imaging tools currently being used in Vietnam include FP and OCT. The expert consensus by APOIS is particularly beneficial in the Vietnamese context, where the availability of ICGA is limited in most clinical settings owing to its invasive, expensive, time-consuming nature, and requirement of specific equipment. Hence, FP and OCT can be considered good alternative to ICGA for the diagnosis of PCV in Vietnam.

### 4.1. Indocyanine Green Angiography

**Figure 2:**
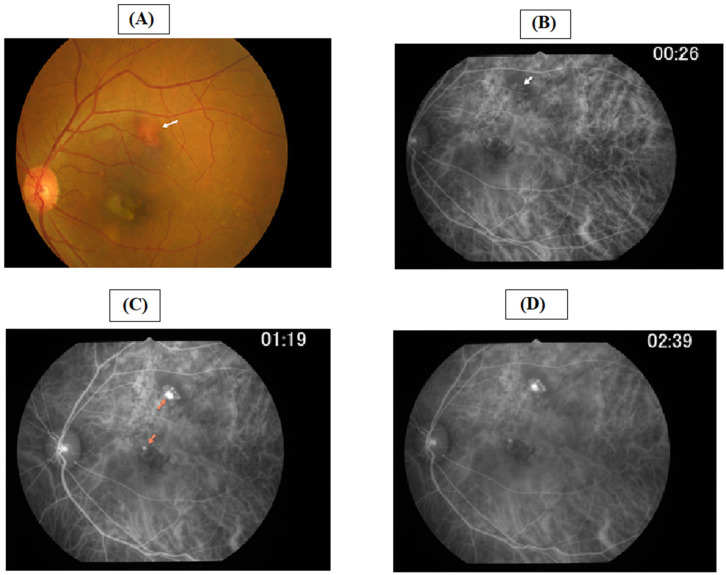
A representation of ICGA in a 56-year-old Vietnamese male patient with PCV in the left eye ((**A**) shows one large orange-red nodule in upper temporal vessel arcade (arrow); (**B**) depicts ICGA early phase showing branching vascular network (white arrow); (**C**) represents ICGA mid-phase showing hot spots (orange arrows) and branching vascular network; (**D**) shows ICGA late phase showing hot spots).

The use of ICGA was established in the early 1990s, when it was used to determine choroidal vascular abnormalities [38]. ICGA is particularly useful in determining the clinical findings of serosanguinous maculopathy and it enables visualization of total lesion area in PCV [39]. A retrospective study was conducted among 40 eyes of Chinese patients with PCV used ICGA as a diagnostic tool. The images obtained described the angiographic characteristics of inactive polypoidal lesions in PCV [40]. With recent digital advancements, ICGA is conducted in combination with a fundus camera or confocal scanning laser ophthalmoscope [41]. However, its use is restricted due to its high cost, invasive nature, requirement for special equipment, and the risk of anaphylactic reaction. [39,42,43]. In Vietnam, the use of ICGA is based on the EVEREST study and the Japanese Study Group of Polypoidal Choroidal Vasculopathy [24]. A survey of Vietnamese ophthalmologists revealed only three ICGA machines being used in Vietnam, suggesting its limited use [22]. Figure 2 shows the presentation of ICGA in a 56-year-old Vietnamese male patient with PCV in the left eye. 

### 4.2. Fundus Photography

**Figure 3:**
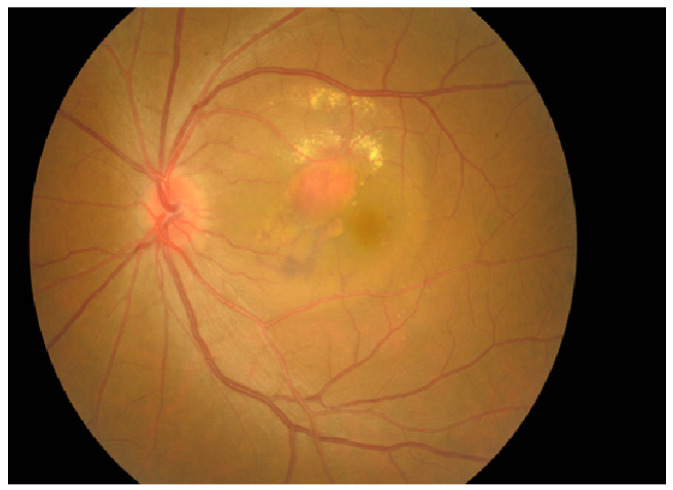
A 51-year-old Vietnamese male patient with PCV with a typical orange-red nodule, hard exudates, and fluid around the nodule in the left eye.

Another important diagnostic tool for PCV is FP, which detects clinical manifestations, including orange nodule, hemorrhagic or fibrovascular PED, massive subretinal hemorrhage, or no/few drusen in the fellow eye, and peripapillary and multifocal lesions [44]. A retrospective study evaluating diagnostic methods used for PCV suggested a sensitivity of 0.73, specificity of 0.82, and area under the receiver operating characteristic curve (AUC) of 0.77 for identifying notched or hemorrhagic PED in patients with PCV [44]. In Vietnam, 43.2% of medical facilities use FP to diagnose PCV [22]. This technique is either used alone or in combination with other techniques such as ICGA or OCT, although it has been observed that FP offers higher specificity compared to other techniques [6,12,42,44]. The use of FP and OCT in combination is of particular importance in differential diagnosis in cases of typical AMD or central serous chorioretinopathy [42]. It also evaluates sensory neuroretinopathy and monitors patients undergoing treatment [12]. In Vietnam, typical signs of PCV could be observed in only half of the patients using FP [24]. Figure 3 depicts an image from a 51-year-old Vietnamese male patient with PCV with a typical orange-red nodule, hard exudates, and fluid around the nodule in the left eye.

### 4.3. Fluorescein Angiography

The typical PCV feature detected using fluorescein angiography (FA) includes an occult choroidal neovascularization leakage pattern, although it has a lower sensitivity and specificity compared with FP and OCT [44]. Additionally, it is evident that the addition of FA with FP and OCT does not improve the accuracy of PCV diagnosis [42]. The determination of leakage of the polypoidal lesions or branching vascular network could be determined using FA, thus proving its use for prognostic purposes [42,45]. The retinal pigment epithelium (RPE) and serosanguinous complications of PCV hamper the visualization of the branched vascular network in FA, thereby restricting its use [39].

### 4.4. Optical Coherence Tomography

The OCT technique can be used alone or in combination with other imaging techniques, thereby assisting the ophthalmologists in diagnosing PCV. It offers higher sensitivity and specificity compared to other techniques. Sharp-peaked PED (sensitivity: 0.94; specificity: 0.79; AUC: 0.86), multilobulated or notched PED (sensitivity: 0.92; specificity: 0.89; AUC: 0.90), hyper-reflective ring below the PED (sensitivity: 0.77; specificity: 0.95; AUC: 0.86), and a double-layered sign (sensitivity: 0.79; specificity: 0.90; AUC: 0.85) are common PCV features identified with OCT [12,42,46]. Subretinal and/or intraretinal fluid could be imaged using OCT, and hence, it finds applicability in monitoring disease activity and therapy response. Additionally, it is applicable for use in clinical settings where ICGA cannot be used. It could also be used to differentiate PCV from wet AMD to a certain extent [3]. As a noninvasive technique, OCT has been utilized in tissue imaging for architectural morphology up to the glandular level [47]. However, artifacts and auto-segmentation limit the usage of OCT [39]. In Vietnam, 88.7% of ophthalmologists chose OCT to diagnose PCV, and >90% of them believed OCT to be the most reliable technique for PCV diagnosis, when IGCA is not available [22]. 

**Figure 4:**
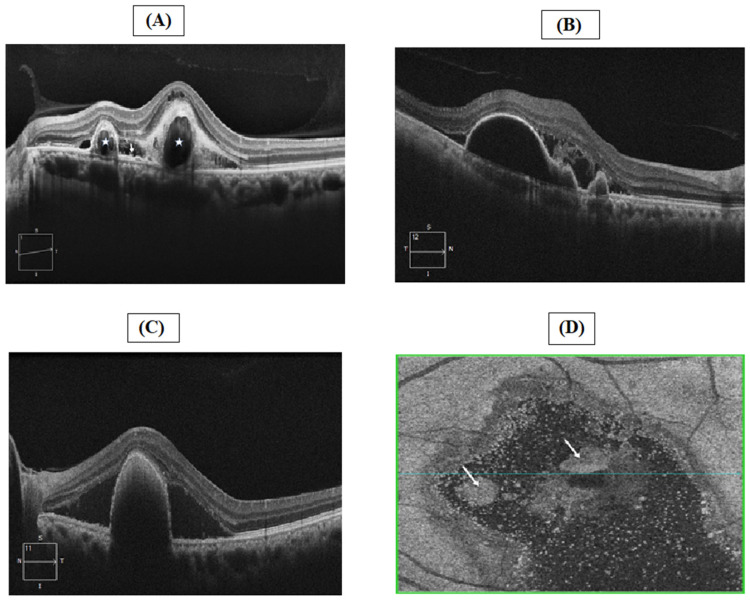
OCT images in eyes with PCV ((**A**) sharp-peaked PEDs with ring shaped (white stars), double-layer sign (white arrow), subretinal fluid and thicken choroid; (**B**) Multiple lobular PED (white arrows), subretinal fluid and hemorrhage [high reflective material]; (**C**) A sharp peak PED with hyper-reflective material at the top underneath the RPE; (**D**) En face OCT: retinal elevation (white arrow)).

Different types of OCT are used for diagnosing PCV. Spectral domain (SD) OCT is a useful technique to differentiate PCV from occult choroidal neovascularization by visualizing coronal scans, allowing for the effective diagnosis of patients presenting with PED [46]. Recently, a systematic review and meta-analysis were conducted to evaluate the diagnostic value of SD-OCT in the diagnosis of PCV. The results show a pooled sensitivity, specificity, and AUC of 0.91, 0.88, and 0.95, respectively. The results demonstrate the high diagnostic value of SD-OCT in PCV diagnosis [48]. Another study conducted among 188 eyes of patients with active PCV used SD-OCT to determine PED, double-layer sign, and thumb-like polyps with a sensitivity and specificity of 89.4% and 85.3%, respectively [49]. Lately, SD-OCT devices have allowed for the visualization of transverse coronal scans (C-scans), which are also called “en face” images. “En face” images differ according to the position of the coronal scan into the retina or choroid. Using the automatic retinal segmentation of the device to define the depth level, two different “en face” OCT images could be generated: outer retinal and choroidal “en face” images. In PCV, a hyper-reflective area of elevated RPE, underlying the branching vascular network, could be observed using this technique [50]. In Vietnam, it was found that SD-OCT is available in 72% of hospitals for the diagnosis of PCV [22]. Figure 4 represents the OCT images in eyes with PCV.

Furthermore, by using enhanced-depth imaging OCT (EDI-OCT), choroidal thickness can be determined and used to differentiate PCV from AMD, where the choroid is usually thin or patients present without choroidal hyperpermeability [51,52]. 

Optical coherence tomography angiography (OCTA) is a newly developed method that can visualize chorioretinal circulation without dye injection. It can detect branching vascular networks clearly, but polyps are not visualized in a few PCV cases. A retrospective study conducted in Taiwan used OCTA as a diagnostic tool to highlight clinical features and treatment response of PCV. The detection and classification of branching vascular networks were also undertaken using OCTA [53]. A study conducted in Vietnam detected OCTA in 60.7% of cases with PCV. This is a useful tool for monitoring disease progression or recurrence [54]. “En face” OCTA may be useful for understanding the pathogenesis of PCV and managing it [50]. A systematic review and meta-analysis by Wang et al. reported a promising role of OCTA in PCV diagnosis, as demonstrated by an AUC of 0.87 (0.84–0.90) [55]. The branched vascular network (BVN) can also be identified easily due to its high flow characteristics. However, the polypoidal lesion detection rate can be highly variable, between 17% and 92% [45], due to slow and turbulent flow within the lesion, the polyps being too small or covered by BVN blood flow signals, or the curvature of the vessels, making it challenging to capture red blood cell motion [45,55]. Other OCTA disadvantages include segmentation errors of peaked PED in PCV that require experienced use of slabs. The images are prone to common artifacts such as motion projection and masking.

The shortcomings of OCTA may be partially compensated for by using swept-source OCT (SS-OCT). SS-OCT has a longer wavelength (1060 mm) and therefore penetrates the pigment epithelium better and, with a high scanning speed of 100,000 scans per second, provides a better resolution of the choroidal structure [56]. The choroidal scleral interface was better visualized, hence providing a more accurate measurement of choroidal thickness, which differentiates PCV from typical nAMD [45]. Recent data show that SS-OCTA can detect up to 90% of polyps [57]. The high image quality of SS-OCTA also provides better BVN structure visualization. A study by Azar showed that a “coral bush-shape” BVN is a positive sign of active type C PCV [56]. However, there was no correlation between disease activity or response to treatment between BVN configuration and PCV type (A, B, or C). These data suggest the ongoing role of ICGA for prognostic and diagnostic values. A new method for visualizing choroidal structure using SS-OCTA called choroidal vasculography (CVG) could improve the rate of PCV diagnosis and evaluate the response to treatment [58].

As Wang et al. recommend in their systemic reviews, a PCV diagnosis can be made if OCTA can identify polyps and BVN. ICG A is necessary only if BVN is detected [55]. This practice will address the bulk of the ICGA burden in clinical practice.

### 4.5. Discussion and Expert Opinion

Diagnostic tools, in line with international guidelines, could be adopted in the guidelines of Vietnam, wherever possible. Although ICGA is regarded as the gold standard in the diagnosis of PCV, its applicability is restricted in Vietnam due to its invasive nature, high cost, and the need for specific equipment. From a Vietnamese perspective, OCT and FP are important diagnostic tools owing to their high accuracy and noninvasive nature. Further, they have been recommended by Vietnam MoH and international associations as an alternative to the ICGA technique. However, in Vietnam, there is a need to evaluate the applicability and utility of different imaging techniques for PCV diagnosis in the absence of ICGA. We recommend using fundus photography, SD-OCT, and SD-OCTA (SS-OCTA if applicable) as standards for multimodal imaging. These noninvasive methods can be used for initial diagnosis and repeated for subsequent follow-ups if needed.

## 5. Treatments

The primary treatment goal for PCV includes improved visual outcomes and the resolution of maculopathy to achieve the best possible vision for patients, as well as reduced treatment burden, thereby improving the quality of life of patients [12,59]. The decision on the type of treatment is based on the disease severity and location of polypoidal lesions. Clinically active symptomatic PCV (presence of intraretinal/subretinal fluid, sub-RPE/subretinal hemorrhage, or vision loss of >5 letters) is often treated. On the other hand, treatment of inactive PCV is based on the physician’s discretion. Typically, only monitoring is required for this group of patients [3,23]. 

**Figure 5:**
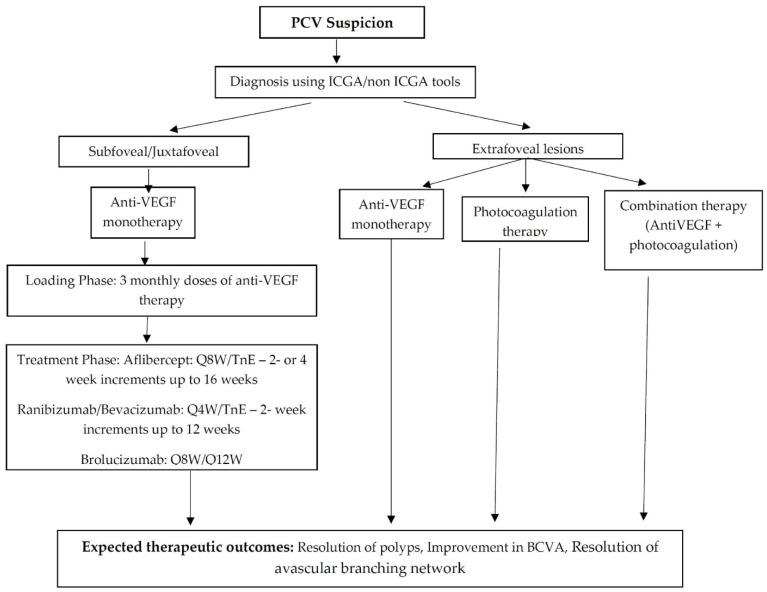
Proposed treatment algorithm for PCV based on latest evidence tailored for Vietnam.

Currently available treatments in Vietnam include photocoagulation lasers and anti-VEGF agents [12]. According to the guidelines of the Vietnamese MoH, PCV can be treated with anti-VEGF as the first line of therapy, similar to nAMD. Although PDT is relatively effective and safe for the management of PCV, it is not currently used in Vietnam [24]. Photocoagulation for extrafoveal lesions may be attempted for patients who cannot afford anti-VEGF treatment or who are unable to follow-up. Figure 5 proposes a treatment algorithm for PCV based on the latest evidence tailored for Vietnam.

### 5.1. Photocoagulation Laser

Photocoagulation laser is widely used in Vietnam to treat retinal diseases, including PCV. The advantage of photocoagulation laser is that it is inexpensive and does not require continuous treatment. On an average, fewer retreatments are required for laser therapy as observed from different studies (1.11–1.33) [59,60]. In Vietnam, only 7.5% of ophthalmologists chose laser photocoagulation to treat patients with PCV [22]. In this method, thermal burns lead to tissue coagulation, leading to improved retinal oxygenation [61]. Photocoagulation therapy is associated with an increased risk of new polypoidal lesions, recurrent bullous detachment, chorioretinal anastomosis, and the occurrence of subretinal hemorrhage leading to vitreous hemorrhage [3]. The long-term effects and safety of laser therapy were established: 69.2% of eyes demonstrated regressed polyps, while 55.6% had a recurrence of polyps during a follow-up period of 72.3 ± 31.0 months. About 15.4% of patients with PCV also exhibited mild subretinal hemorrhage [62]. In a study conducted in Vietnam, 53.1% of patients reported a reduced 0.3 log MAR (logarithm of the minimum angle of resolution) during the six-month follow-up post-photocoagulation treatment [24]. In a separate study in the Chinese population, 56% of patients with PCV reported stable or improved vision with laser therapy [21]. Similarly, the efficacy of conventional argon laser was established at 12 months in a retrospective analysis wherein 36% of patients reported improved vision (logMAR improvement > 0.2 units), 39% had stable vision (logMAR change ≤0.2 units), while 25% experienced decreased vision (logMAR decrease >0.2 units). Further, 64.3% of patients had complete resolution of maculopathy [59]. To avoid the risk of hemorrhage or scotomas, a photocoagulation laser is most useful for treating lesions of smaller sizes, such as extrafoveal/peripheral/peri-papillary polypoidal lesions [3]. Of note, 70% of Vietnamese patients with PCV had lesions located in the macula [24], and hence, the applicability of photocoagulation laser therapy is limited in Vietnam.

### 5.2. Anti-VEGF Therapies

Anti-VEGF therapy has revolutionized the treatment of PCV and other vitreoretinal diseases. The pathophysiological rationale for the use of anti-VEGF therapy is based on the outcomes of clinical studies, showing an increased level of VEGF in the aqueous humor and its positive correlation with active PCV [63,64]. The advantages of anti-VEGF include treating polypoidal lesions at or outside the macular area, not requiring complicated equipment, and the ability to use without performing ICGA. There are currently three anti-VEGF drugs available for clinical use in Vietnam: aflibercept, ranibizumab, and bevacizumab (off-label usage). Brolucizumab will be launched in Vietnam soon [12,65]. Efficacy, cost, and availability are the key factors driving the selection of anti-VEGF drug. The percentage of hospitals in Vietnam with access to bevacizumab, ranibizumab, and aflibercept is 70.5%, 70.5%, and 30.1%, respectively [22]. The common anti-VEGF regimens used in Vietnam are treat-and-extend and pro re nata [22]. 

In the recent guidelines, anti-VEGF is recommended as monotherapy or in combination with PDT for the treatment of PCV. A meta-analysis conducted on the use of anti-VEGF therapy for PCV established the superiority of the combination therapy of PDT and anti-VEGF, in terms of best-corrected visual acuity (BCVA) improvement, complete polyp regression, and decrease in central retinal thickness—especially in early combination therapy [66]. However, due to the nonavailability of PDT in Vietnam, anti-VEGF is currently used as monotherapy. 

#### 5.2.1. Bevacizumab

Bevacizumab was the first anti-VEGF to be used in Vietnam, although it has not been approved by the Vietnamese MoH for the indication of ocular diseases. However, due to its low cost, it is widely used as an off-label drug for the treatment of PCV and nAMD in Vietnam [12]. In a study of 16 patients evaluating the efficacy of intravitreal bevacizumab injection monotherapy in PCV, the mean foveal thickness decreased along with a slight improvement in mean visual acuity from 0.54 ± 0.38 to 0.45 ± 0.32 in logMAR after three months. However, this subsided and was reversed after 12 months [67]. A study conducted in the Vietnamese population demonstrated that 54.5% of patients had vision loss and only 9.1% had increased vision after six months of treatment, which contrasted with the results of a previous study conducted among patients with typical nAMD, in which bevacizumab showed good efficacy in improving visual acuity [24,25].

#### 5.2.2. Ranibizumab

Ranibizumab is a full-length humanized VEGF antibody fragment that neutralizes all biological isoforms of VEGF-A and has been approved for the treatment of nAMD [68]. It is the first anti-VEGF drug to be approved for use in ocular diseases by the Vietnamese MoH. Several studies have evaluated and established the efficacy of ranibizumab in the treatment of PCV. In a prospective, open-label trial where monthly intravitreal 0.5-mg ranibizumab injections (IVRs) were administered to 12 patients with PCV; no patients lost ≥15 letters in visual acuity after six months. Preliminary results exhibited stabilized vision, resolution of subretinal hemorrhage (100%), and improved macular edema (80%); also 33% of the patients had reduced polypoidal lesions [68]. In a randomized clinical trial (LAPTOP study), where patients were either treated with PDT or IVR of 0.5 mg, 30.4% of patients reported improved visual acuity, 60.9% experienced no change, and 8.7% had reduced acuity with IVR treatment. The outcome was significantly better for IVR treatment in comparison to the PDT therapy. Additionally, improvement in retinal thickness (418.9 ± 168.6 μm to 311.2 ± 146.9 μm, *p* < 0.001) and logMAR (0.48 ± 0.27 to 0.39 ± 0.26, *p* < 0.003) was also observed [69]. In a Japanese observational study conducted among 45 eyes with PCV and treated with IVR, 87% of eyes showed improvement in BCVA after 24 months. However, the need for retreatment was observed in eyes with serous retinal detachment [70]. A prospective, observational study conducted in Taiwan among 161 treatment-naïve patients reported an improvement in the mean gain in BCVA and a decrease in mean central retinal thickness. However, 58.4% of patients reported adverse events, and 98.8% were deemed not related to study treatment [71].

The studies were conducted to compare the effectiveness of IVR monotherapy in comparison to the combination of IVR and PDT. The multicenter double-masked EVEREST trial including 61 Asian patients established that PDT in combination with IVR or alone, was superior in terms of complete polyp regression compared with IVR monotherapy (77.8% and 71.4% vs. 28.6%; *p* < 0.01). The change observed in terms of BCVA (letters) was 10.9 ± 10.9 (PDT + IVR), 7.5 ± 10.6 (PDT alone), and 9.2 ± 12.4 (IVR monotherapy), indicating a better outcome with IVR monotherapy compared to PDT alone. There were no safety concerns with any of the treatment regimens [72]. A double-masked, multicenter, randomized clinical trial, the EVEREST II trial (involving 322 Asian patients) compared the effectiveness and safety profiles of combination therapy (PDT and IVR) and IVR monotherapy. The superiority of combination therapy compared to IVR monotherapy was evident in terms of BCVA (letters) (8.3 vs. 5.1) and complete polyp regression (69.3% vs. 34.7%; *p* < 0.001). A higher dosage frequency was observed with IVR monotherapy (seven injections) compared with combination therapy (four injections). However, vitreous hemorrhage was the only serious ocular side effect observed with both therapies [73]. 

In a meta-analysis conducted to determine the practicability of IVR compared with PDT, it was established that logMAR visual acuity shifted from 0.6 to 0.3 and the improvement rate in the visual acuity was 60% to 70% in the IVR group over a duration of 24 months. However, PDT demonstrated improvement in visual acuity (35%) over a short-term follow-up period [74]. Guidelines have recommended the use of IVR in combination with PDT for treating PCV. Since PDT is unavailable in Vietnam, the clinical utility of combination therapy of PDT and IVR would be limited; further, this limitation can act as a barrier to ranibizumab therapy. 

#### 5.2.3. Aflibercept

Aflibercept is a VEGF trap developed using fusion protein and is primarily used to suppress the concentration of VEGF in the aqueous humor [75,76]. It is a decoy receptor that binds all the isoforms of VEGF-A, VEGF-B, and placental growth factors. It has a higher affinity for VEGF (Kd~1 pM) than VEGF receptor, bevacizumab, or ranibizumab for VEGF, suggesting a long duration of biological activity. Furthermore, time-dependent mathematical models predicted prolonged (10–12 weeks) intravitreal VEGF-binding activity for aflibercept compared to four weeks of biological activity for ranibizumab. This unique mechanism of action beyond VEGF-A, and the longer duration of activity, provides potential advantages compared to other anti-VEGF agents. This leads to reduced angiogenesis and vascular permeability, thereby providing a longer duration of action than other anti-VEGFs, including ranibizumab [77,78,79]. From a patient perspective, this could mean fewer injections and physician visits, lower costs, a smaller risk from intravitreal injections, fewer drug-related adverse events, and improved compliance. 

The efficacy of aflibercept as monotherapy in PCV has been demonstrated in numerous studies. A prospective, open-label, single-arm multicenter clinical trial (APOLLO study) conducted among patients with PCV using intravitreal aflibercept injection (IVA) (2.0 mg) as monotherapy showed that BCVA at one year was either maintained or improved in 97.6% of patients and that the mean logMAR BCVA improved to 0.12 logMAR compared to baseline (0.33 logMAR). Reduction in central foveal thickness and complete regression of polypoidal lesions (72.5%) were also evident in IVA treated patients [80]. Similar results were obtained in a phase IV, prospective, single-arm, interventional case series (VAULT study), where at 12 months of treatment, BCVA was maintained in 87.5% of treated eyes, central subfield macular thickness significantly reduced compared to baseline, 66.7% of eyes showed complete polyp regression, and the macula was dry in 60% of eyes. These favorable outcomes were, however, seen along with fluid recurrence in 33% of treated eyes with significantly lower vision gain at 12 months PCV [81]. 

A prospective, multicenter, double-masked, sham-controlled, randomized clinical study (PLANET study) was conducted to compare the efficacy of IVA monotherapy and IVA in combination with PDT in patients with PCV. Improvement in visual and functional outcomes was observed in 85% of the patients treated with IVA monotherapy. Only 12.1% of the patients required rescue PDT, and >85% of the patients had favorable visual and functional outcomes with IVA monotherapy [82]. After two years, IVA monotherapy was non-inferior to combination therapy in terms of improving visual acuity (+10.7 vs. +9.1 letters, *p* = 0.48), with a rate of polyp regression achieved of over 80%, which was comparable between both groups. Although 17% of the patients required rescue PDT, more than 80% of the patients responded well to IVA monotherapy [83]. 

Aflibercept has been found to be effective in patients with PCV refractory to ranibizumab by using a treat-and-extend regimen. It results in a significant BCVA improvement, shrinkage of branching vascular networks, a decrease in mean lesion diameter, and polyp regression. Aflibercept was found to be effective in resolution of the retinal fluid in patients with PCV [84,85]. To our knowledge, there are currently a few well-designed randomized controlled studies that have made a head-to-head comparison of anti-VEGF agents in PCV. In a study of 100 eyes with treatment-naïve PCV, IVA was found to be superior to IVR in improving visual acuity after 24 months [86]. In a study conducted among 98 eyes with treatment-naïve PCV, the rate of polyp regression in the IVA group was superior to that of IVR (39.5% vs. 21.6%; *p* = 0.007) [87]. In a retrospective analysis of 101 eyes of 101 patients with PCV conducted in Taiwan, IVA as monotherapy was as effective as IVR plus PDT in improving visual acuity and anatomical outcomes [88]. 

The Asia-Pacific Vitreo-retina Society recommended use of aflibercept according to the treat-and-extend regimen to provide personalized care to patients with PCV [89]. 

#### 5.2.4. Brolucizumab

Brolucizumab is a novel antibody fragment used for the management of nAMD; preclinical studies have suggested effective tissue penetration and better intraretinal, subretinal, and subretinal pigment epithelium fluid control across retinal layers [90]. The phase 3 HAWK and HARRIER trials have established a similar well-tolerated safety profile and superior anatomical outcomes for brolucizumab compared with aflibercept. The measured parameters were LS mean in BCVA (+6.6 [6 mg] and +6.1 [3 mg] letters with brolucizumab vs. +6.8 letters with aflibercept [HAWK]; +6.9 letters with 6 mg brolucizumab vs. +7.6 letters with aflibercept [HARRIER]; *p* < 0.001), retinal thickness (HAWK −172.8 μm vs. −143.7 μm; HARRIER −193.8 μm vs. −143.9 μm), retinal fluid changes, and safety [90,91]. In the Japanese cohort of the HAWK study involving 69 PCV patients, brolucizumab was found to be as effective as aflibercept in terms of visual acuity improvement, superior with respect to fluid resolution, and had a favorable anatomical outcome in 76% of brolucizumab-treated patients. However, the risk of intraocular inflammation was higher in the brolucizumab group [92]. The post hoc analysis of the HAWK/HARRIER study also confirmed the occurrence of retinal vasculitis with or without retinal vascular occlusion, along with an augmented risk of visual acuity loss using brolucizumab injections [93]. A higher incidence of retinal vasculitis and intraocular inflammation were also observed in a retrospective case series, with clinical manifestations in the form of focal or elongated segments of sheathing, optic nerve swelling, Kyrieleis plaques, retinal whitening from arterial occlusion, cotton-wool spots, paracentral acute middle maculopathy, intraretinal hemorrhages, vitreous cells or opacities, and late perivenular hemorrhages. This often causes a worsening of visual acuity, leading to vision loss [94]. 

A recent study in Japan compared the short-term outcomes of three-monthly IVA and brolucizumab injections in 52 treatment-naïve PCV eyes and showed comparable outcomes across the two treatments in terms of BCVA improvement (from 0.27 ± 0.34 [log MAR unit] at baseline to 0.20 ± 0.24 after 3 months) and reduction (43–44%) in central retinal thickness. However, the brolucizumab-treated group was superior to IVA in terms of decrease in subfoveal choroidal thickness and the complete resolution rate of polypoidal lesions (78.6% vs. 42.1%). Notably, intraocular inflammation was observed in 14.3% of patients in the brolucizumab-treated group only [95]. Several health agencies, including the Food and Drug Administration and European Medicines Agency, have designed brolucizumab as a second-line option after other anti-VEGFs have failed due to its associated adverse events [96]. 

### 5.3. Discussion and Expert Opinion

Treatment approaches in line with international guidelines could be incorporated into the Vietnamese guidelines, if feasible. Currently, anti-VEGF monotherapy is the first choice for PCV treatment in Vietnam. The most common treatment regimen used in Vietnam for PCV includes monthly bevacizumab injections adapted to a treat-and-extend approach, albeit as an off-label therapy. Monthly bevacizumab injections help in controlling exudation but seem to fail to regress polyps. Recurrent subretinal hemorrhages and subretinal pigment epithelium hemorrhages are frequently occurring adverse events after one to two years of use, even in stable cases. Additionally, bevacizumab affordability is an important factor determining its use in Vietnam. Currently, ranibizumab is covered by health insurance and has been used in Vietnam for PCV management. In general, ranibizumab combined with PDT is the optimal option, but in Vietnam, ranibizumab monotherapy could help control disease progression. Due to its favorable functional and anatomical outcomes, aflibercept should be the preferred drug of choice if anti-VEGF monotherapy is considered. The lower treatment burden of aflibercept compared to ranibizumab and bevacizumab provides an additional advantage. The high price can be a barrier for patients; however, the relative merits of more expensive drug with a prolonged activity and less frequent dosing versus a more frequently dosed, lower-cost alternative, such as off-label bevacizumab, need to be carefully considered by retinal physicians. For this reason, it is necessary to provide a cost–benefit analysis for these treatments to improve patient access. Healthcare centers in Vietnam need to create awareness about novel anti-VEGF therapies and hold patient education programs about PCV. Laser photocoagulation is an option if patients with extrafoveal lesions cannot afford or follow-up with anti-VEGF treatments. However, the use of this therapy should only be considered when polypoidal lesions are outside of the macular region. Combination therapy may be considered in multifocal polyps to reduce the burden of treatment, primarily for anti-VEGF and laser photocoagulation combinations.

## 6. Conclusions

The strength of this article lies in the fact that it is the first review article to emphasize the complete PCV landscape in Vietnam, from clinical perceptions to therapies. The increasing prevalence of PCV requires ophthalmologists to carefully screen patients for better management and provide adequate counseling for improved patient compliance and adherence to therapy. Individualized treatment regimens might prove beneficial on a case-by-case basis, and such scenarios might be highlighted for future reference. Furthermore, to address current needs or gaps, PCV-related guidelines in Vietnam should be in alignment with international guidelines, wherever applicable and possible.

## Data Availability

Unpublished data from a national survey in Vietnam that support the narrative discussions presented in this manuscript are available from the corresponding author, Dang Tran Dat, upon reasonable request.
